# Integration analysis of miRNA–mRNA expression exploring their potential roles in intrahepatic cholangiocarcinoma

**DOI:** 10.1038/s41598-023-35288-0

**Published:** 2023-05-24

**Authors:** Liyan Liu, Yajun Shi, Pengjie Zhang, Xing Zhang

**Affiliations:** 1 Department of Blood Transfusion, The Affiliated Changzhou No. 2 People’s Hospital of Nanjing Medical University, Changzhou, People’s Republic of China; 2 Institute for Fetology, The First Affiliated Hospital of Soochow University, Suzhou, People’s Republic of China; 3 School of Chemistry and Life Science, Suzhou University of Science and Technology, Suzhou, People’s Republic of China

**Keywords:** Microbiology, Pathogenesis

## Abstract

Intrahepatic cholangiocarcinoma (ICC) is the second common primary hepatic malignancy tumor. In this study, an integrative analysis of differentially expressed genes (DEGs) and miRNAs from the ICC onset and adjacent normal tissues were performed to explore the regulatory roles of miRNA–mRNA interaction. A total of 1018 DEGs and 39 miRNAs were likely involved in ICC pathogenesis, suggesting the changes in cell metabolism in ICC development. The built network indicated that 30 DEGs were regulated by 16 differentially expressed miRNA. The screened DEGs and miRNA together were probably considered the biomarkers of ICC, and their important roles in ICC pathogenesis remain to be elucidated. This study could provide a good basis to uncover the regulatory mechanism of miRNA and mRNAs in ICC pathogenesis.

## Introduction

Cholangiocarcinoma (CCA) is a type of cancer that forms in the slender tubes and is classified into the rare intrahepatic cholangiocarcinoma (ICC 20–25%), and perihilar (50–60%) or common distal bile duct tumors^1,2^. This aggressive cholangiocarcinoma arises from the malignant proliferation of epithelial cells located proximal to the second-degree bile ducts^3^. Following hepatocellular carcinoma (HCC), it is still reported as the second most common primary hepatic malignancy accounting for 5% to 30% of all primary hepatic cancers^4^. The clinical ICC presentation appears generalized abdominal pain, or less commonly weight loss and jaundice. Owing to the nonspecific symptoms of ICC and its relatively rare incidence, it is difficult to diagnose ICC at an early stage and the epidemiology of ICC remains ambiguous^1,5^. Cross-sectional imaging with either computed tomography or magnetic resonance imaging hardly aids in the initial diagnosis. Thus, the transcriptional analysis would be helpful to understand prognostic features.

The microRNAs (miRNAs) are small non-coding RNAs with lengths of 18–22 nucleotides, regulating gene expression in a post-transcription manner^6,7^. They demonstrated great potential in diverse physiological and pathological processes^8^. The abnormal miRNA expression may elicit tumor-suppressive or oncogenic consequences and lead to the physiological and pathological development of many cancers^9^. To date, hundreds of miRNAs have been brought out in tumor development, invasion, metastasis, and drug resistance. Besides, miRNAs have been identified as a biomarker for the diagnosis and prognosis of different cancers^10^. The ever-increasing observations evidence the critical role of the regulatory network of miRNA–mRNA in different cancer types^11–17^. Currently, there are some new understandings of the roles of miRNAs in the pathogenesis of the ICC. The exosomal miR-3124-5p can target growth differentiation factor 11 (GDF11) and reduce its expression, which promotes the malignant progression of ICC. Transcription factor YY1-induced DLEU1/miR-149-5p promotes the malignant biological behavior of ICCs via upregulation of YAP1, TEAD2, and SOX2. The miR-196-5p can boost cell proliferation and migration in ICC pathogenesis via HAND1/Wnt/β-catenin signaling pathway^18^. These results demonstrated that miRNAs as important regulatory non-coding RNAs played pivotal roles in the progress and pathogenesis^19^. However, there are fewer reports to insight into the regulatory miRNA–mRNA networks in ICC pathogenesis.

In this study, we sequenced the expression of mRNAs and miRNAs simultaneously and conducted an integrated analysis to explore the functional interaction networks of miRNA–mRNA in ICC. The insight into the regulatory miRNA–mRNA networks would be of importance to glance at the gene expression in ICC pathogenesis.

## Materials and methods

### Patient tissues preparation and RNA extraction

The resected ICC tissues and adjacent normal tissues were collected from curative surgey for ICC patients hospitalized at the First Affiliated Hospital of Soochow University during the period of February 2018 to October 2020. The Samples of ICC tissues (ICC1, ICC2, and ICC3) and adjacent normal tissues (Nor1, Nor2, and Nor3) were from three different patients at the TNM II stage according to the condition of tumor development (Table S1). This study was approved by the Ethics Committee of the First Affiliated Hospital of Soochow University (No.2021-335) and the experiments with human samples confirmed to the guidelines set by the Declaration of Helsinki. Written informed consent was obtained from participants before their enrollment in the study. For RNA extraction, all samples of ICC and adjacent normal tissues were quickly frozen in liquid nitrogen after resecting. The total RNAs for whole transcriptome sequencing were extracted using Trizol reagent (Invitrogen). The total RNAs for small RNA sequencing were extracted using the mirVana miRNA Isolation Kit (Ambion). An Agilent 2100 Bioanalyzer (Agilent Technologies) was applied to the evaluation of RNA integrity, and the samples with an RNA integrity number (RIN) ≥ 7 were used for the subsequent analysis.

### Transcriptome sequencing and analysis

The rRNA-depleted and strand-specific RNA library constructions were constructed using TruSeq Stranded Total RNA with Ribo-Zero Gold (Illumina). The constructed libraries were sequenced on the HiSeqTM 2500 sequencing platform (Illumina) by OE Biotech (Shanghai, China). The raw reads were quality filtered o get high-quality reads. Trimmomatic software was used to remove adapters. The clean reads were aligned to the reference genome of humans (GRCh38.p12) by hisat2. the read counts were normalized by using the estimateSizeFactors function of the DESeq (2012) R package, and *P* values and fold changes were calculated by its nbinomTest function. The criteria for differentially expressed genes (DEGs) were set as adjusted *P* values < 0.05 and fold changes ≥ 2. The GO annotation and KEGG enrichment were performed by using R based on a hypergeometric distribution test.

### Small RNA sequencing and analysis

To detect the small RNA expression patterns in ICC tissues, small RNA libraries were generated by using TruSeq Small RNA Sample Preparation Kits (Illumina). Small RNA sequencing was conducted by OE Biotech (Shanghai, China). The basic reads were converted into raw reads by base calling. The clean reads were obtained by filtration of the read neither shorter than 15 nt nor longer than 41 nt. The known miRNAs were identified by alignment with miRBase v.22 (http://www.mirbase.org/)^20^. The unannotated small RNAs were mined with mirdeep2^21^. Similarly, the differentially expressed miRNAs were calculated with the DEG algorithm with the thresholds of *P* values < 0.05 and fold changes ≥ 2. The targets of miRNAs were predicted with miRanda software with the parameters of ΔG ≤ − 30 kcal/mol and strict 5′ seed pairing^22^. GO annotation and KEGG enrichment were performed by using R based on a hypergeometric distribution test^23–25^.

### miRNA–mRNA interaction network construction and GO/KEGG analysis

The normalized read counts of DEGs and differentially expressed miRNAs were extracted and used to construct miRNA–mRNA regulatory networks according to the negatively correlated miRNAs and mRNAs. GO annotation and KEGG enrichment analysis of differentially expressed miRNA with their target genes were performed using R based on the hypergeometric distribution test^23–25^.

## Results

### Analysis of the mRNA expression pattern in ICC tissues

**Figure 1:**
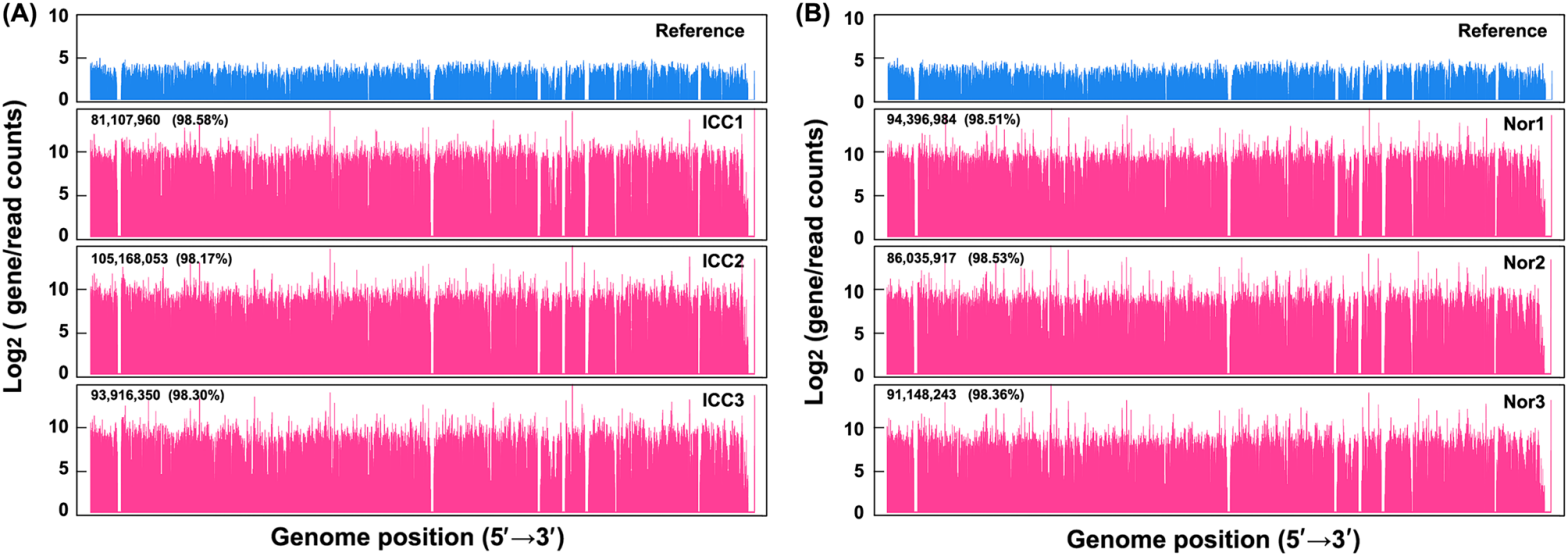
Distribution of the sequencing reads for the samples of (**A**) ICC tissues and (**B**) adjacent normal tissues. The gene distribution in the reference genome (GRCh38.p12) was shown in blue.

**Figure 2:**
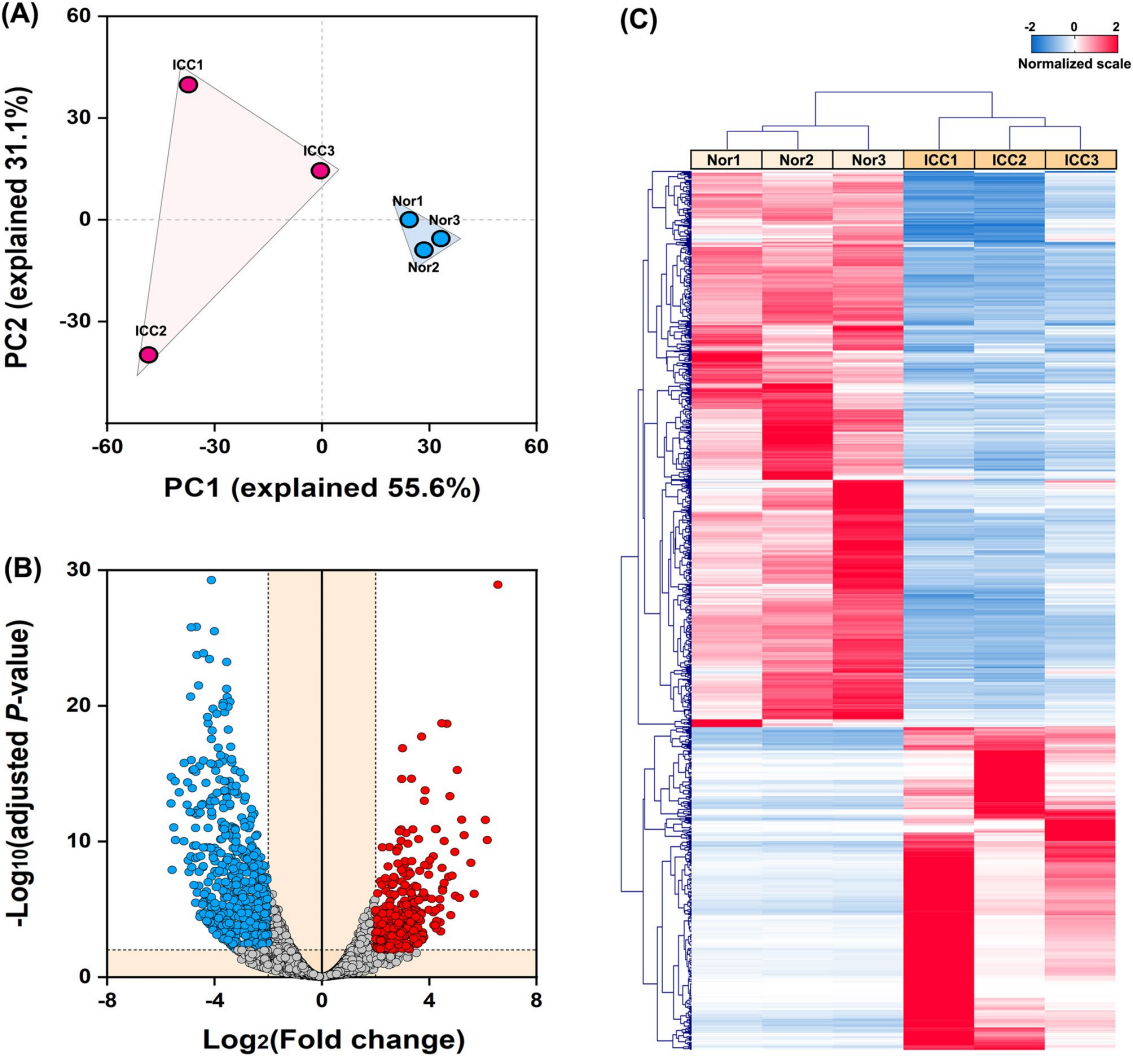
Analysis of the mRNA expression pattern in ICC tissues. (**A**) PCA analysis of the samples of ICC tissues (ICCs) and the adjunct normal tissues (Nors). Two groups were differentiated by shadows. (**B**) Volcano plot of gene expression. The reference lines of − Log_10_(adjusted *P*-value) and |Log_2_(fold change)| were set as 2. The down-regulated and up-regulated DEGs were shown in blue and red. (**C**) Heatmap of the DEGs. All samples were clustered into two groups (ICCs and Nors).

To uncover mRNAs expression pattern in the ICC development and progression, three samples from the ICC tissues (ICC1, ICC2, and ICC3) was analyzed with RNA sequencing against the adjacent normal tissues (Nor1, Nor2, and Nor3). All the raw data were deposited into the public database with accession numbers PRJNA763019 and PRJNA763017. An average of 94,982,664 and 91,935,189 clean reads were obtained from the samples of ICCs and Nors, respectively. The mapped reads to the reference genome (GRCh38.p12) account for more than 98% of the total reads from both groups(Table S2). The read distribution indicated good sequencing depth and coverage (Fig. 1). The principal component analysis (PCA) suggested a distinct and diversified gene expression pattern in the ICC tissues in comparison with the adjacent normal tissues (Fig. 2A). To analyze gene expression patterns, the DEGs from ICCs against Nors were visualized with the volcano plot. The criterion of fold change ≥ 2.0 and adjusted *P* value ≤ 0.05 filtered out 947 up-regulated genes and 1,298 down-regulated genes, whereas the up-regulated and down-regulated gene numbers were lessened to 374 and 644, respectively, by using a strict criterion of fold change ≥ 4.0 and adjusted *P* value < 0.01 (Fig. 2B and Table S3). These DEGs were hierarchically clustered in an unsupervised model, and two samples were separated in the heatmap (Fig. 2C).

### KEGG pathway and GO enrichment analyses of DEGs in ICC tissues

**Figure 3:**
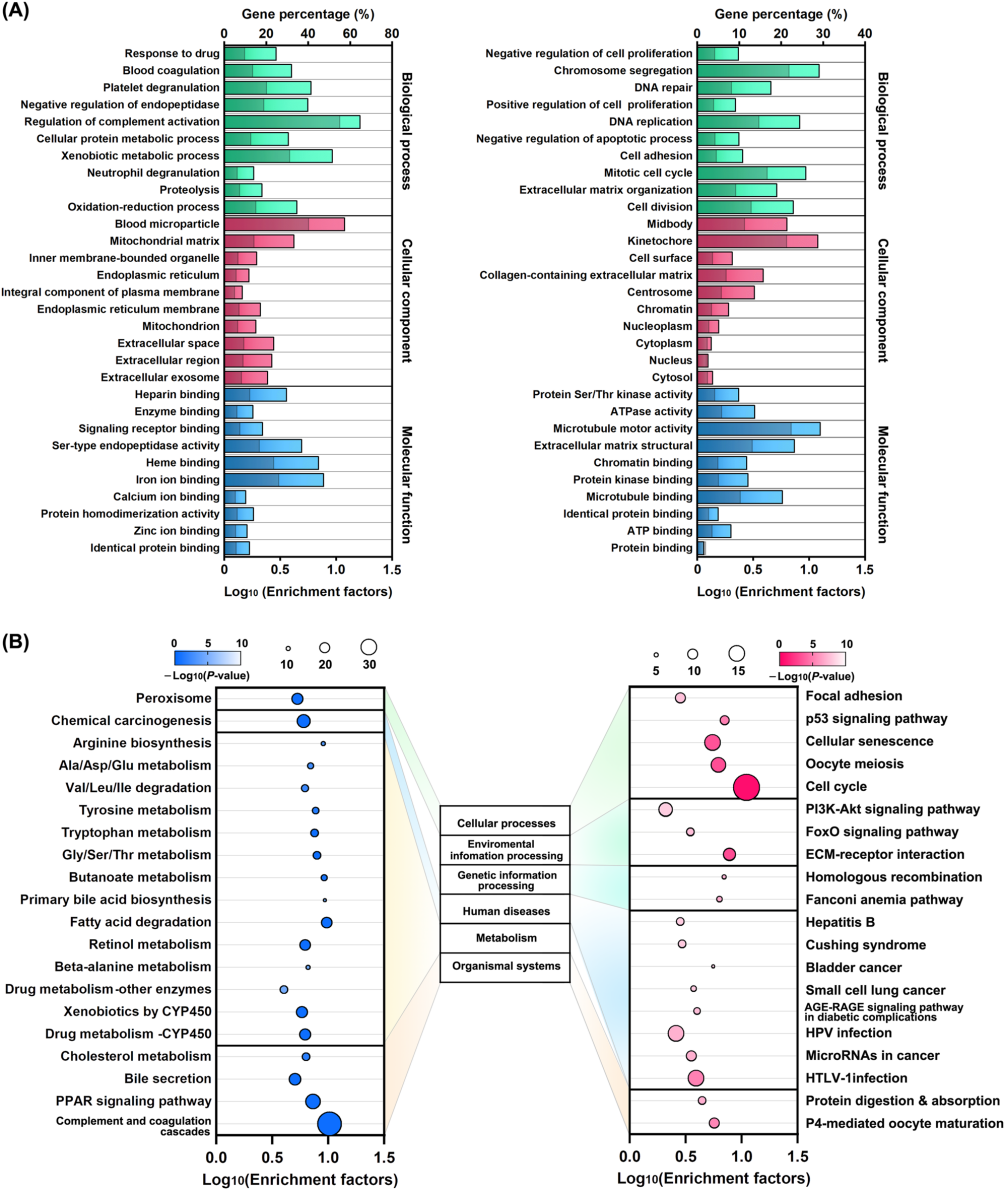
Analyses of (**A**) GO Enrichment and (**B**) KEGG Pathway of DEGs in ICC tissues. The down-regulated (left panel) and Up-regulated DEGs (right panel) were analyzed for GO terms and KEGG pathways, respectively. GO terms were shown in green, red, and blue for different categories. The gray bars indicate enriched DEGs per the background genes of each GO term.

To clarify the potential function of the DEGs in ICC tissue, the up-regulated and down-regulated DEGs were subject to GO analysis and KEGG enrichment analysis, respectively. The top 10 GO terms from categories of biological process, cellular component, and molecular function were shown in Fig. 3A. The Up-regulated DEGs were mainly associated with cell growth and proliferation in the biological process. many structural cellular components such as the nucleus, cytoplasm, and chromatin were significantly enriched. Among the top 10 molecular functions, protein binding, ATP binding, and microtubule binding were significantly (*P* ≤ 0.05) enriched. On the contrary, the down-regulated DEGs were mainly associated with cell responses and their communications in biological processes. The components of extracellular exosomes, endoplasmic reticulum, and peroxisomes were significantly enriched, and the molecular functions included activity modulation by small compound binding. The KEGG analysis revealed 30 and 56 metabolic pathways for the enrichment of the up-regulated and down-regulated DEGs, respectively. The top 20 pathways were categorized into the cellular process, environmental information processing, genetic information processing, human diseases, metabolism, and organismal systems. To note, many pathways associated with metabolisms of amino acids and xenobiotics were repressed, and the activated pathways were related to several human disease factors, including microRNAs (miRNA) in cancer (Fig. 3B). Of the enriched gene set (BRCA1, CCNE2, CDCA5, CDKN2A, E2F1, E2F2, KIF23, SOX4, TGFB2, TRIM71; 10/202 of hsa06206), breast cancer type 1 susceptibility protein BRCA1 specifically mediates the formation of Lys-6-linked polyubiquitin chains and plays a central role in DNA repair by facilitating cellular responses to DNA damage, E3 ubiquitin-protein ligase TRIM71 can behave the post-transcriptional mRNA repression in a miRNA independent mechanism. The E3 ubiquitin-protein ligase activity is required for its tumor suppressor function, and all these enriched genes are engaged in miRNA interactions for the development of some cancers. Altogether, these results revealed that ICC development did change cell metabolism, and the DEG pattern was probably linked to miRNA regulation.

### Analysis of the miRNA expression pattern in ICC tissues

**Figure 4:**
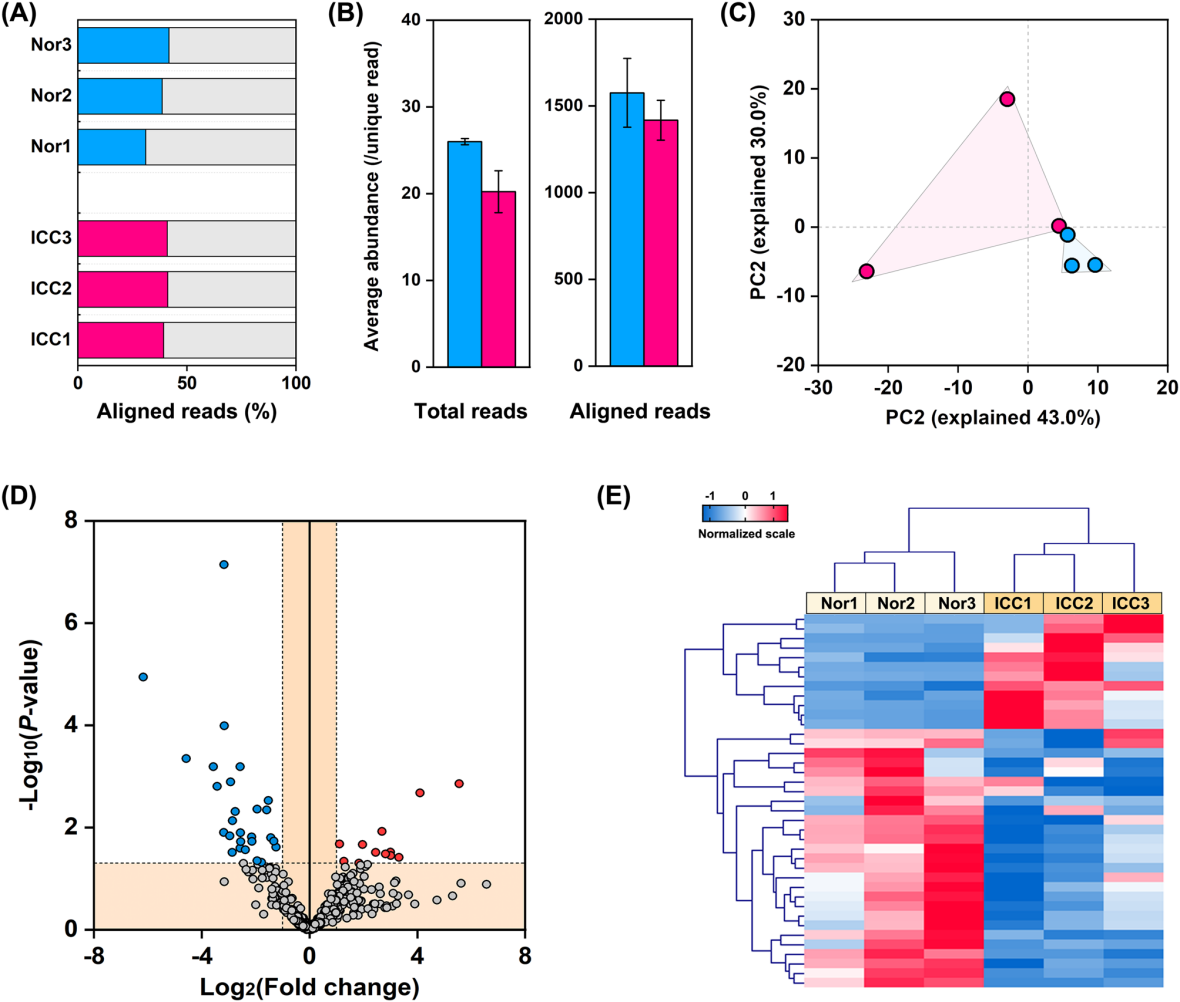
Analysis of the miRNA expression pattern in ICC tissues. (A) Percentage and (**B**) abundance of the aligned reads for the samples of ICC tissues (ICCs) and the adjunct normal tissues (Nors). (**C**) PCA analysis of the samples (ICCs and Nors), the groups were differentiated by shadows. (**D**) Volcano plot of miRNA expression. The reference lines of − Log_10_(adjusted *P* value) and |Log_2_(fold change)| were set as 1. The significantly down-regulated and up-regulated miRNA was shown in blue and red. (**E**) Heatmap of the differentially expressed miRNA. All samples were clustered into two groups (ICCs and Nors).

To identify the differentially expressed miRNAs in ICC tissues, small RNA libraries were constructed from the samples of ICC tissues. The clean reads were in a range of 13,741,533–15,498,480 in both ICCs and Nors, and more than 92.1% could be mapped to the reference genome of humans. To note, around 30–40% of reads were aligned to miRbase version 22.0 in both samples (Fig. 4A), and their average abundance was dozen times higher than that of the total reads for both samples (Fig. 4B). The PCA plots showed a distinct miRNA expression pattern of the ICCs to that of the Nors, which was in line with the observation of their mRNA expression patterns (Fig. 2B). A total of 521 aligned (or predicted) miRNAs with baseMean of ≥ 10 were filtrated at the criterion of fold change ≥ 2.0 and *P* value < 0.05 to screen the deferentially expressed miRNAs. As a result, there were 12 significantly up-regulated and 27 significantly down-regulated miRNAs (Fig. 4C, and Table S4). Within an unsupervised model, these differentially expressed miRNAs could be grouped according to the sample source (Fig. 4D), and they were also clearly separated in the heatmap (Fig. 4E).

### Target prediction for the differentially expressed miRNAs and function analysis

**Figure 5:**
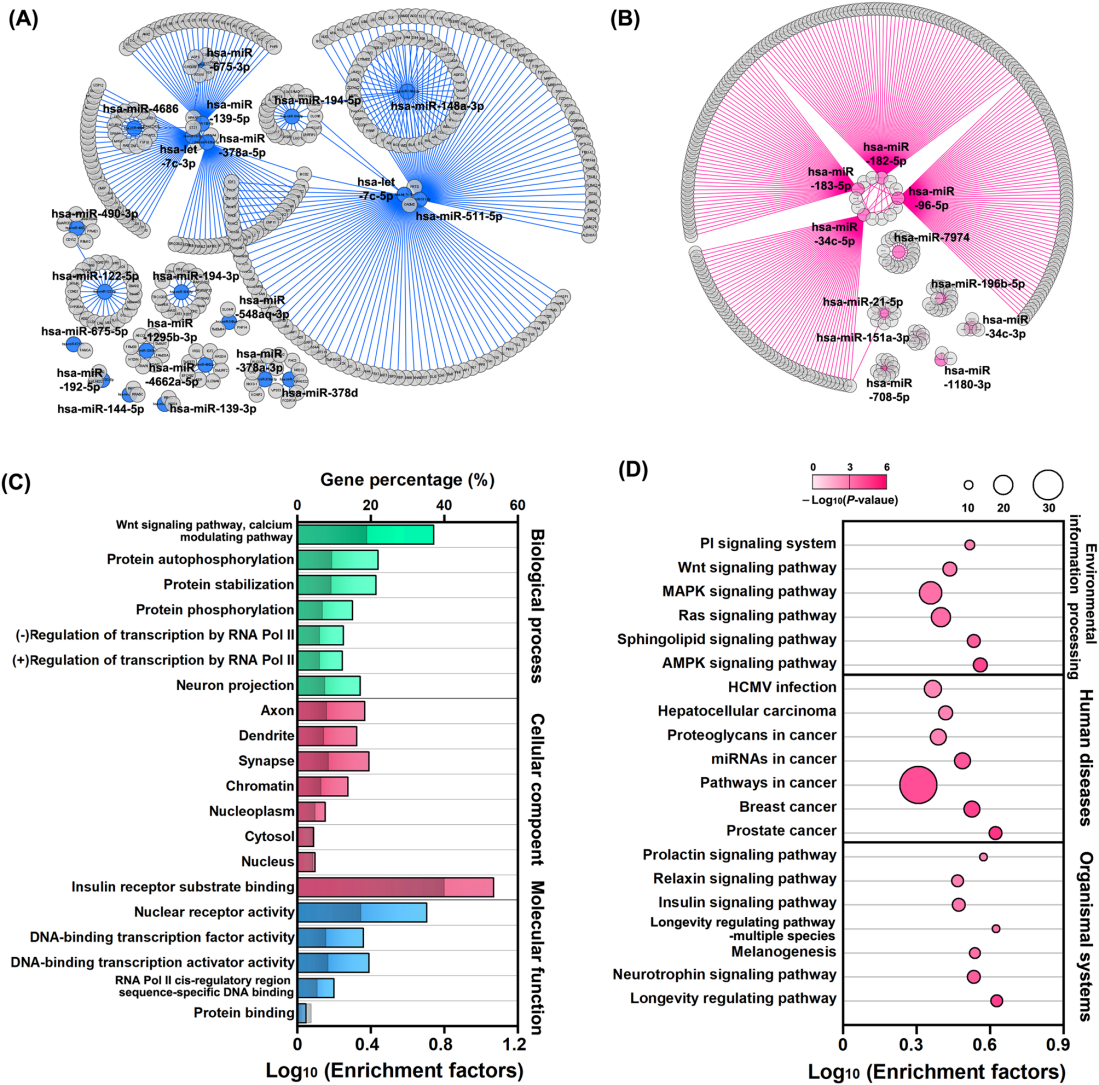
Target prediction for the differentially expressed miRNAs and function analysis. Target prediction for significantly (**A**) down-regulated and (**B**) up-regulated miRNAs. The predicted genes surrounding the differentially expressed miRNA are shown in grey. The analyses of (**C**) GO Enrichment and (**D**) KEGG Pathway were performed with all (615) the predicted targets. The gray bars indicate enriched targets per the background genes of each GO term.

To understand the potential roles of the differentially expressed miRNAs in ICCs, their target mRNAs were predicted with miRanda algorithms (Table S5). Twenty-one down-regulated miRNAs were predicted to regulate 365 targets via 380 interactions, and eleven up-regulated miRNAs probably interplay with 309 predicted targets via 322 interactions (Fig. 5A,B). These targeted candidates were further subjected to GO terms and KEGG enrichment analysis. The GO enrichments indicated that the critical biological process, cellular component, and molecular function focused on the Wnt signaling pathway, calcium modulating pathway, and those related to cell proliferation, axons, synapses, and protein stabilization (Fig. 5C). The KEGG pathway analysis revealed that these targeted genes were categorized into environmental information processing, human disease, and organismal system (Fig. 5D). Many pathways are correlated to cancer development, hinting at the crucial roles in the ICC pathogenesis.

### Integrative analysis of miRNA and DEGs expression profiling in ICC tissues

**Figure 6:**
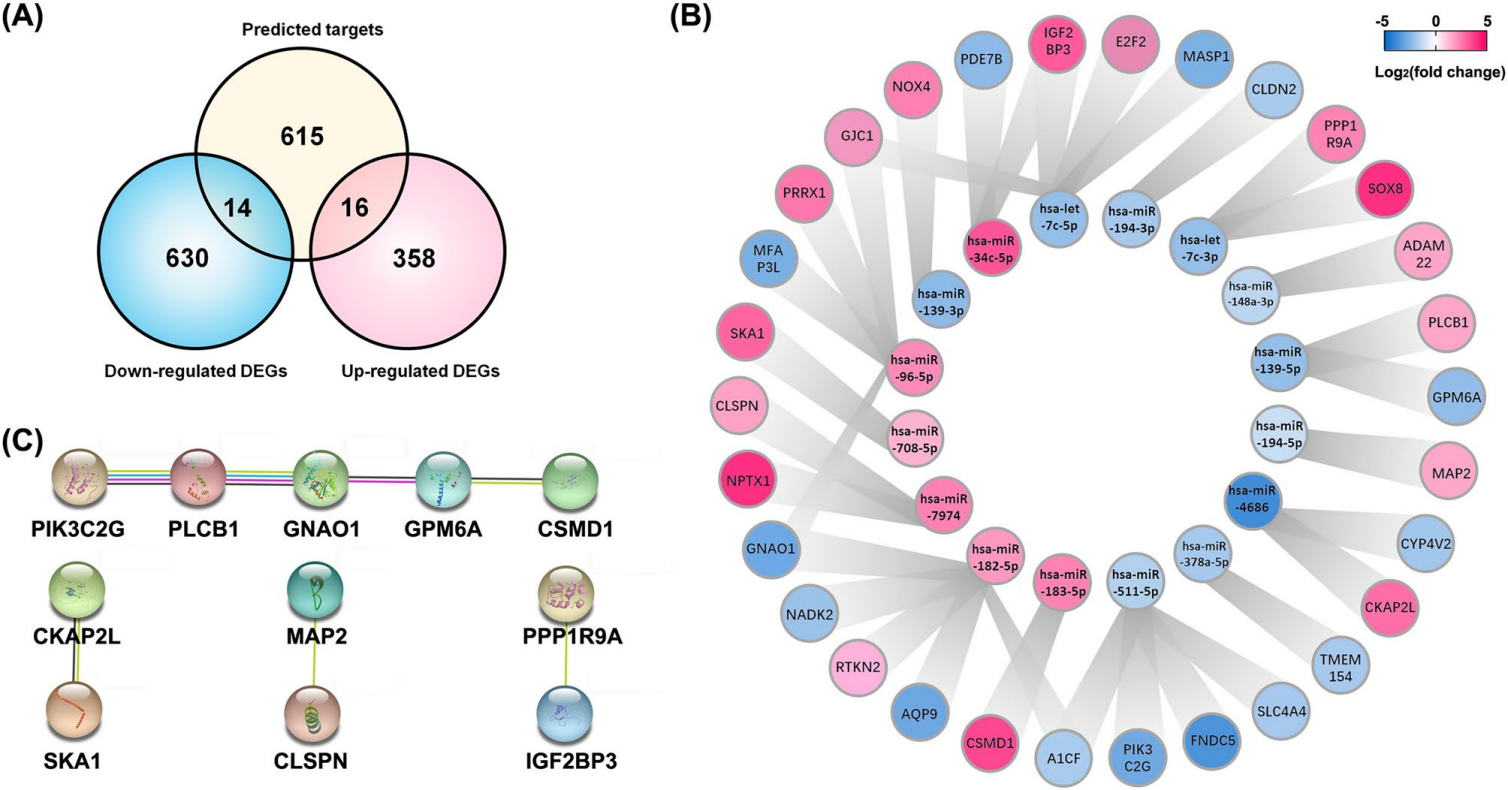
Integrative analysis of miRNA and DEGs expression profiling in ICC tissues. (**A**) Venn diagram of the DEGs and predicted targets. (**B**) Regulation network of the 30 screened DEGs with the relevant miRNAs. The down-regulation and the up-regulation of DEGs and miRNAs were shown in blue and red, respectively. (**C**) String analysis (string-db.org, Version 11.5) for protein–protein interaction of the 30 screened DEGs.

The intersection genes between the predicted targets for differentially expressed miRNAs and the DEGs were visualized in the Venn diagram (Fig. 6A). There were 16 up-regulated DEGs (RTKN2, IGF2BP3, PLCB1, PPP1R9A, SOX8, MAP2, PRRX1, E2F2, CKAP2L, CLSPN, GJC1, NPTX1, NOX4, ADAM22, CSMD1, and SKA1) and 14 down-regulated DEGs (AQP9, A1CF, CLDN2, CYP4V2, GNAO1, PIK3C2G, MFAP3L, GPM6A, TMEM154, PDE7B, FNDC5, MASP1, SLC4A4, and NADK2) overlapped with predicted targets, respectively. They were the targets of 16 miRNAs (hsa-miR-7974, hsa-let-7c-3p/-5p, hsa-miR-139-3p/-5p, hsa-miR-148a-3p, hsa-miR-378a-5p, hsa-miR-4686, hsa-miR-511-5p, hsa-miR-182-5p, hsa-miR-183-5p, hsa-miR-96-5p, hsa-miR-34c-5p, hsa-miR-194-3p/-5p, and hsa-miR-708-5p), constituting 34 interactions (Fig. 6B). The string analysis suggested that 11/30 proteins are likely to form 7 edges with a protein–protein interaction *P* value of 0.0206 (Fig. 6C). Thus, these 30 targets mediated by the miRNAs may play pivotal roles in either progression or pathogenesis of ICC and remain to elucidate.

## Discussion

In this study, the integrative analysis of mRNA and miRNA expression in ICC was performed with transcriptome sequencing. The potential functions of DEGs and differential miRNAs were comprehensively explored to understand the miRNA–mRNA regulatory mechanism on the ICC pathogenesis and progression. There are 1018 DEGs identified from the ICC tissues, suggesting the aberrant transcriptional expression and their crucial roles in the ICC etiology and pathogenesis. Functional and KEGG analysis showed that these DEGs s were related to functions of cell proliferation, protein metabolisms, and extracellular matrix structural constituent, as well as many human diseases-relevant signaling pathways. Some genes could be further screened as prognostic markers of the ICC. Moreover, the upregulated DEGs were enriched in pathways in cancer, human papillomavirus (HPV) infection, and PI3K-Akt signaling pathway, whereas the downregulated DEGs were enriched in complement and coagulation cascades, chemical carcinogenesis, peroxisome, PPAR signaling pathway, and retinol metabolism. Living habits would be a crucial contributing etiologic factor for chronic liver disease including ICCs and obesity, especially viral hepatitis and ICC^26^. Many CCA risk factors are common to the ICC, such as cirrhosis, viral hepatitis, alcohol use, and tobacco use. Obesity, hypertension, and glucose and lipid metabolism disorder, are also crucial etiologic factors for cancers^1,26–28^. We guessed that factors like alcohol use, chemical contamination, and metabolic syndrome could be the shared risks for ICC and other liver diseases.

Molecular pathway level analysis found that the dysregulated expression of DEGs in ICC was associated with the metabolism of aberrant tRNA, amino acid, and lipoprotein^29^. The increasing pieces of evidence also revealed that aberrantly expressed miRNAs were common in various types of human cancer and play important roles in tumorigenesis^30–33^. Thank to the high-throughput sequencing of small RNAs, 39 differentially expressed miRNAs were filtrated from the ICC tissues. The clustering analysis showed the distinct expression profiles of miRNAs between ICC tissues and adjacent normal tissues, suggesting a potential role of these miRNAs as biomarkers for ICC diagnosis and therapeutic targets. It was to note that cholangiocarcinoma was originated from the epithelium of the bile duct, and most cancerous tissue was consisted adenocarcinoma cells from bile duct^34–36^. Such an aberrant expression profile of miRNA–mRNA might be related to bile duct lesions. Given the analyzed ICC specimens were at TNM II stage, the clinical stage of perhaps also contributes to this observation. The miRNA expression of miR-433, miR-22, miR‐21, miR-125b-5p, miR-551b-3p, and miR-182 have been reported to regulate the progress or pathogenesis of ICC via regulating target genes expression^37–40^. For example, miR-182 was maily expressed in ductular reaction cells and was associated with cholangiocyte damage and ductular reaction. Knockdown of miR-182 resulted in reduced bile acid accumulation and cholestasis, even reduced live injury and inflammation^41^. In this regard, It was hypothesized this proposed miRNA/mRNA network might be relevent to bile acid levels, cholestasis, and inflammation. Besides, The presence of hepatitis virus (e.g. HCV) infection would reinforce in ICC progression^32^, whereas our results did not suggest HCV infection is involved in ICC progression. As none analyzed case suffered from HCV infection in this study, it remained to elucidate its contribution by sampling the HCV infected cases.

The present study also predicted the miRNA–mRNA interaction in ICC pathogenesis via GO term and KEGG analysis. Of 39 miRNAs, 32 candidates were predicted to regulate 615 targets, which were involved in the Wnt signaling pathway, calcium modulating pathway, and those related to cell proliferation, axons, synapses, and protein stabilization. These results demonstrated that differentially expressed miRNAs may function as a crucial regulator in ICC pathogenesis by modulating the target genes. The regulation was also founded on the important amino acid and fatty acid metabolic pathways, which was in line with the previous observation^29,42^. But be aware that the analyzed samples were collected from surgical resection rather than laser capture microdissection, so the moderately precise separation of ICC specimens would cause condemination of epithelial cells from stromal cells, and increase the background noise to discriminate the clonal origins of ICC^43–46^. Moreover, ICC cells interact with a complex milieu of supporting stromal cells that form the tumor microenvironment. single cell atlases provide important insight into understanding the contribution of different cell clusters to the malignant process in a spatial view^46^. Such an analysis distinguish the clonal origins of the subtupes of the combined HCC-ICCs^47^. Therefore, a more comprehensive biological and clinical insight into ICC could be excepected by sampling operation with aser capture microdissection and futher integration of signle cell atlases. In conclusion, the present study suggested a set of mRNAs and miRNAs involved in ICC pathogenesis by integrative analysis of their expression profiles in the ICC tissues against adjacent normal tissues. It would be a good case to uncover the regulatory mechanism of miRNA and mRNAs in ICC pathogenesis, which could assist us with development of new therapies for ICC.

## Supplementary Information

Supplementary Tables.

## Data Availability

Raw sequence reads for mRNA (Accession: PRJNA763017) and miRNA (Accession: PRJNA763019) were deposited in NCBI database. Other data are available from the corresponding author upon reasonable request.
